# Improved outcomes from the administration of progesterone for patients with acute severe traumatic brain injury: a randomized controlled trial

**DOI:** 10.1186/cc6887

**Published:** 2008-04-30

**Authors:** Guomin Xiao, Jing Wei, Weiqi Yan, Weimin Wang, Zhenhui Lu

**Affiliations:** 1 Department of Neurosurgery and Neurotrauma Center, Affiliated Hospital, College of Medicine, Hangzhou Normal University, Hangzhou 310015, China; 2 Department of Health Center, Affiliated Hospital, College of Medicine, Hangzhou Normal University, Hangzhou 310015, China; 3 Clinical Research Centre, Second Affiliated Hospital, College of Medicine, Zhejiang University, Hangzhou 310009, China

## Abstract

**Background:**

Severe traumatic brain injury (TBI) has been increasing with greater incidence of injuries from traffic or sporting accidents. Although there are a number of animal models of TBI using progesterone for head injury, the effects of progesterone on neurologic outcome of acute TBI patients remain unclear. The aim of the present clinical study was to assess the longer-term efficacy of progesterone on the improvement in neurologic outcome of patients with acute severe TBI.

**Methods:**

A total of 159 patients who arrived within 8 hours of injury with a Glasgow Coma Score ≤ 8 were enrolled in the study. A prospective, randomized, placebo-controlled trial of progesterone was conducted in the Neurotrauma Center of our teaching hospital. The patients were randomized to receive either progesterone or placebo. The primary endpoint was the Glasgow Outcome Scale score 3 months after brain injury. Secondary efficacy endpoints included the modified Functional Independence Measure score and mortality. In a follow-up protocol at 6 months, the Glasgow Outcome Scale and the modified Functional Independence Measure scores were again determined.

**Results:**

Of the 159 patients randomized, 82 received progesterone and 77 received placebo. The demographic characteristics, the mechanism of injury, and the time of treatment were compared for the two groups. After 3 months and 6 months of treatment, the dichotomized Glasgow Outcome Scale score analysis exhibited more favorable outcomes among the patients who were given progesterone compared with the control individuals (*P *= 0.034 and *P *= 0.048, respectively). The modified Functional Independence Measure scores in the progesterone group were higher than those in the placebo group at both 3-month and 6-month follow-up (*P *< 0.05 and *P *< 0.01). The mortality rate of the progesterone group was significantly lower than that of the placebo group at 6-month follow-up (*P *< 0.05). The mean intracranial pressure values 72 hours and 7 days after injury were lower in the progesterone group than in the placebo group, but there was no statistical significance between the two groups (*P *> 0.05). Instances of complications and adverse events associated with the administration of progesterone were not found.

**Conclusion:**

Our data suggest that acute severe TBI patients with administration of progesterone hold improved neurologic outcomes for up to 6 months. These results provide information important for further large and multicenter clinical trials on progesterone as a promising neuroprotective drug.

**Trial Registration:**

ACTRN12607000545460.

## Introduction

Traumatic brain injury (TBI) remains one of the leading causes of injury-related death and severe disability. The management of TBI currently includes preventing further neurological insults, managing the intracranial pressure (ICP), and surgical procedures. It is very important to search for clinically effective neuroprotective drugs to prevent secondary brain injury after TBI. In spite of many neuroprotective agents showing efficacy in experimental models of TBI, none has produced significant neuronal protection when tested in clinical trials [1,2].

Progesterone, a hormone, has steroidal, neuroactive and neurosteroidal action in the center neuronal system. Neuroprotective effects of progesterone have recently been shown in a variety of animal models, including ischemic and traumatic brain insult models [3-6]. Postinjury administration of progesterone in experimental models of head injury confers significant protection against TBI-induced cerebral edema and secondary neuronal death, promoting behavioral recovery [7,8]. Experimental evidence suggests that postinjury treatment with progesterone decreases brain edema, attenuates free radical damage, and reduces neuronal loss in TBI animal models [8-13]. Progesterone also reduces the inflammatory response and attenuates neurological abnormalities after ischemia and spinal cord injury [14-18].

In a recently published controlled study of progesterone, Wright and colleagues conducted a phase II, randomized, double-blind, placebo-controlled trial to assess the safety and benefit of administering progesterone to patients with acute TBI [19]. No serious adverse events were found in the 77 patients who received progesterone, and the patients with moderate TBI who received progesterone were more likely to have a moderate to good outcome than those were randomized to placebo at 30 days post injury. The 30-day mortality in the progesterone group was less than one-half that of the control group. This outcome suggests that progesterone causes no harms and may be a beneficial treatment for TBI [19].

Despite these potential advantages and the good safety profile of progesterone described in studies utilizing animals or humans as subjects, there is relatively little information available from assessing neuroprotective properties of progesterone in the patients with acute severe brain trauma. The effects of progesterone on neurological outcome of the TBI patients remain unclear. The purpose of the present pilot clinical study was to assess the longer-term efficacy of progesterone on improving the neurological outcome of patients with acute severe TBI.

## Materials and methods

### Patients

Patients with acute severe TBI and a Glasgow Coma Scale (GCS) score ≤ 8 after resuscitation and stabilization were entered into the study. Two hundred and thirty patients from the Neurotrauma Center of our teaching hospital were included. Male or female patients between the ages of 18 and 65 years were studied. The patients received progesterone within 8 hours after the documented time of injury. All patients admitted to the Neurotrauma Center, Clinical Medical College of Hangzhou between March 2004 and February 2007 were consecutively eligible for enrollment.

We excluded patients who had received any investigational drugs 30 days prior to the enrollment, such as progesterone, estrogen and investigational compound, patients with severe anoxic intracerebral damage or brain death, and patients whose clinical condition was unstable (partial pressure of oxygen < 60 mmHg or a systolic blood pressure < 90 mmHg, or both). We also excluded pregnant patients and lactating female patients, and those for whom there was doubt whether the neurological status resulted from head trauma or acute or chronic spinal cord injury.

The study was conducted in compliance with the clinical protocol approved by the Institutional Review Board and the ethical committees of Clinical Medical College of Hangzhou, according to Good Clinical Practice standards. Because of the nature of patients' injuries, subjects in this clinical study were incapable of granting informed consent. Investigators therefore obtained informed consent from the subject's legal guardian or health proxy before administering the drug. Given the urgent circumstances, we were unable to obtain permission from a legal guardian or health proxy within the stipulated time window for some patients (n = 53). Investigators therefore sought approval from the Institutional Review Board to use deferred consent. If the Institutional Review Board determined that these regulatory criteria were satisfied, the investigators were permitted to enroll subjects without consent. When the drug was administered without proxy consent, the Institutional Review Board was notified within 2 working days. We continued to try to contact the proxy consent after drug administration, and documented those attempts to the Institutional Review Board. Once contacted, the family or legally authorized representative was notified of the patient's enrollment and asked to provide written approval for the patient's continued participation. If attempts to contact proxy consent were unsuccessful, or if the patient died before the family could be contacted, we notified the Institutional Review Board and placed a full report in the patient's record and study file.

### Standard clinical management

After head computerized tomography scanning, the patients were delivered to the neurosurgical intensive care unit of the teaching hospital immediately or following surgical evacuation of an intracranial hematoma. All patients received the standard treatment for management of severe TBI based on the guidelines for the management of severe head injury of the American Association of Neurologic Surgeons [20]. Particular emphasis was placed on the prevention and treatment of secondary insults, the avoidance of intracranial hypertension, maintenance of a normovolemic state as well as normothermia and normoglycemia, with ventilation to maintain the oxygen pressure at a minimum of 100 mmHg and the carbon dioxide pressure at approximately 35 mmHg.

### Randomization and medication administration

The prospective, randomized, placebo-controlled, double-blind study was conducted in our neurosurgical intensive care unit. Subjects enrolled in the study were randomized to receive either progesterone (Tianjing Jinyao Pharmaceutical Co. Ltd, Tianjing, China) or matching placebo within 8 hours of the documented time of injury. Qualifying patients were randomly assigned in a 1:1 manner to receive the matching treatment with random numbers. Patients for the treatment group were given progesterone at 1.0 mg/kg via intramuscular injection and then once per 12 hours for 5 consecutive days. A single-dosage volume equivalent to 0.05 ml/kg was used in each subject. In a double-blind manner, progesterone and placebo were supplied via identical-looking solutions in identical glass vials with or without progesterone. The appearance, packaging and administration of placebo and progesterone injections were the same for the two groups. All patients, treating physicians, nursing staff, and pharmacists were blinded throughout the study period.

### Clinical measurements

The ICP was monitored continuously using ICP monitoring apparatus (CAMINO. MPM-1; Integra Co., San Diego, CA, USA). A computerized tomography scan was obtained in all patients at admission and this was categorized according to the modified Marshall computerized tomography scan classification: I, intracranial pathology not visible on the computerized tomography scan; II, cisterns present with shift ≤ 5 mm; lesions present, but no high-density or mixed-density lesions > 25 cm^3^, with bone fragments and foreign bodies; III, cisterns compressed or absent, shift ≤ 5 mm, with no high-density or mixed-density lesions > 25 cm^3^; IV, shift > 5 mm, with no high-density or mixed-density lesions >25 cm^3^; V, any surgically evacuated lesion; and VI, high-density or mixed-density lesions >25 cm^3 ^without surgical evacuation.

The patient's condition – body temperature, heart rate and respiratory rate, blood pressure, and pulse blood oxygen saturation – was monitored continuously at the bedside with monitoring apparatus (Hewlett-Packard, Palo Alto, CA, USA). Daily evaluations of neurologic status over the initial 14-day period were performed via the GCS score, adverse experiences, surgical procedures, and intracranial complications. Intake and output of fluids were also recorded.

Laboratory tests including hematology, the coagulation profile and clinical chemistry were performed daily and then for 1 week after injury. A urine pregnancy test was performed at enrollment for female patients (as necessary).

### Neurologic outcome measurements

The neurologic outcome was evaluated according to the Glasgow Outcome Scale (GOS) score, which contains five levels of outcome: good recovery, moderate disability, severe disability, vegetative survival, or death. For statistical analysis, GOS scores were dichotomized into favorable or unfavorable outcomes. Patients in the upper two GOS outcome groups (good recovery and moderate disability) were considered of favorable outcome, and patients in the other groups (severe disability, vegetative state, or death) were considered of unfavorable outcome.

Secondary efficacy endpoints were the modified Functional Independence Measure (FIM) score and mortality. Based on previous reports [21,22], the modified FIM measurements of disability in three areas of activity (domains of self-care, motor function, and cognitive function) were chosen from the 18 items in the full FIM. Each of three items (expression, feeding, and locomotion) includes four possible levels of function ranging from total dependence (1) to independence (4). The total modified FIM scores therefore ranged from 3 to 12. The patients were assessed using the same measures both at 3 and 6 months in the follow-up protocol.

### Statistical analysis

Descriptive statistics, including proportions, means and standard deviations, were compiled for all demographic and outcome measures. Demographic and clinical data were analyzed using Fisher's exact test. The statistical analyses were conducted to assess the differences between the treatment group and the control group on specific variables. Statistical analysis was performed using contingency analysis (chi-squared) for categorical data and Student's *t *test for continuous data. *P *< 0.05 was considered statistically significant. SPSS 11.0 software package (SPSS Inc., Chicago IL, USA) was used for statistical analysis.

## Results

### Patients

**Figure 1:**
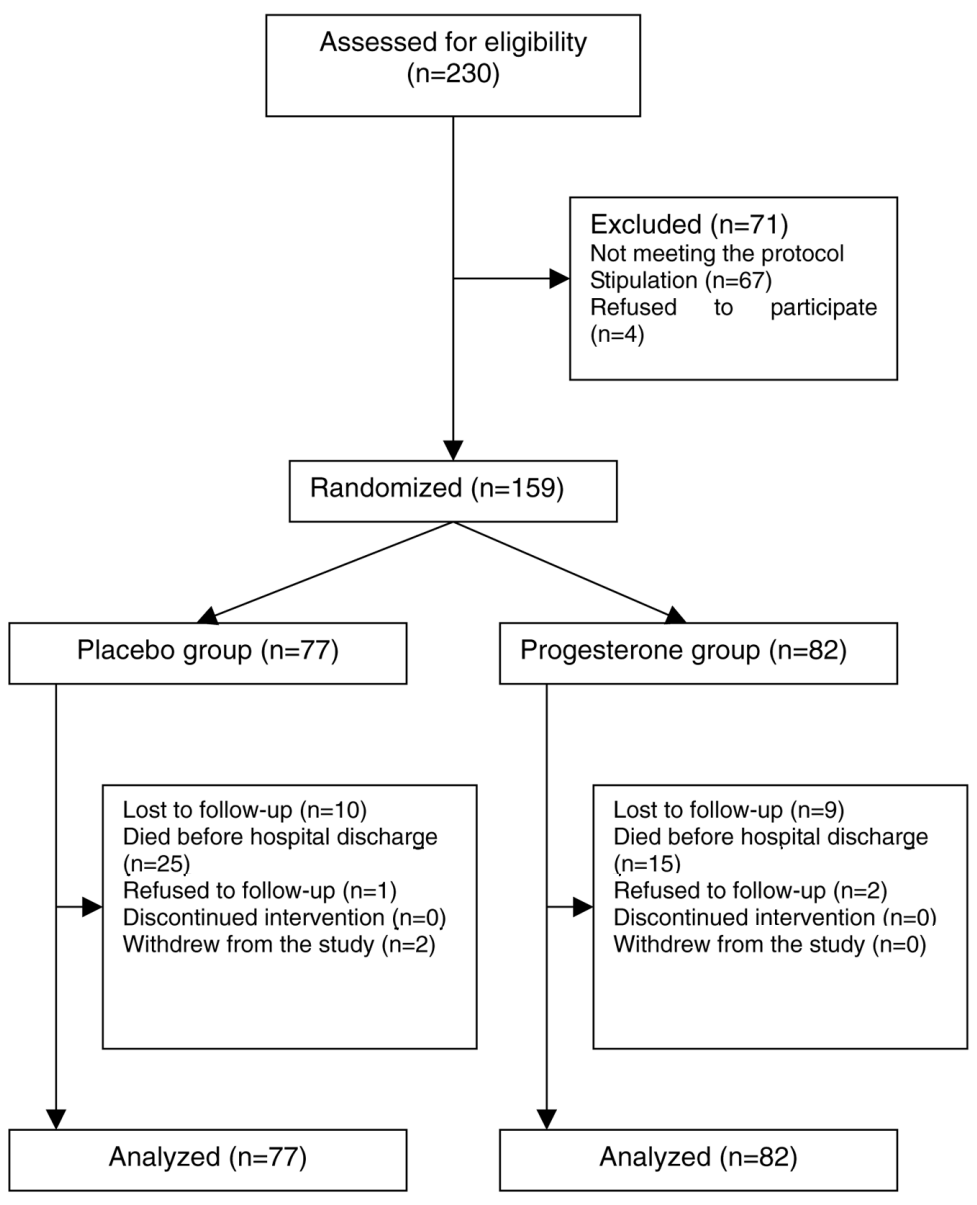
Trial profile.

Between March 2004 and February 2007, a total of 230 patients were screened in the present study. Of these, 159 patients meeting the protocol stipulation and condition were recruited and randomized to receive either progesterone (n = 82) or placebo (n = 77). Data were available for 154 patients (96%) at the 3-month follow-up and for 135 patients (84%) at the 6-month follow-up. Nineteen patients (11%) were lost to follow-up, three patients (1%) refused follow-up, and two patients (1%) withdrew from the trial. No subjects were enrolled in violation of the protocol stipulations (Figure 1).

The demographics of the progesterone and placebo groups are presented in Table 1. The cohorts were well balanced with no significant differences between the two groups. The medication history of patients, medication administration, and medical procedures were not significantly different among treatment groups.

**Table 1:** Clinical and demographic characteristics between the two groups

Admission characteristic	Placebo (n = 77)	Progesterone (n = 82)	*P *value
Males	57 (74)	58 (70)	0.64
Females	20 (25)	24 (29)	0.64
Mean (standard deviation) age (years)	31 (9)	30 (11)	0.52
Mean (standard deviation) time injury to administration (hours)	3.65 (1.46)	3.80 (2.03)	0.59
Mean (standard deviation) qualifying Glasgow Coma Scale score	6.1 (1.3)	6.0 (1.8)	0.68
Glasgow Coma Scale 3 to 5	20 (25)	22 (26)	0.90
Mechanism of injury			
Motor vehicle	62 (80)	63 (76)	0.57
Fall	8 (10)	10 (12)	0.71
Assault	4 (5)	7 (8)	0.40
Other	3 (3)	2 (2)	0.59
Surgical procedures	24 (31)	22 (26)	0.54
Pupillary response			
Bilaterally normal	26 (33)	26 (31)	0.78
Abnormal	51 (66)	56 (68)	0.78
Marshall computerized tomography scan classification			
I	0	0	
II	9 (11)	7 (8)	0.50
III	22 (28)	28 (34)	0.44
IV	13 (16)	12 (14)	0.69
V	24 (31)	22 (26)	0.54
VI	9 (11)	13 (15)	0.44

Data presented as n (%) unless indicated otherwise.

### Glasgow Outcome Scale scores

**Figure 2:**
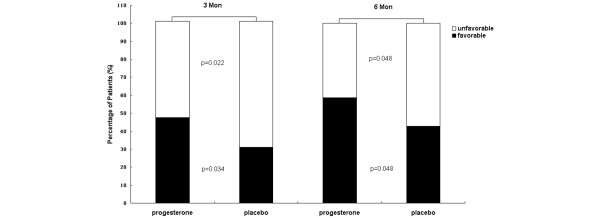
Dichotomized Glasgow Outcome Scale scores for patients receiving either progesterone or placebo. There was a remarkably more favorable outcome among patients who were given progesterone compared with patients receiving placebo (*P *= 0.034) 3 months postinjury. At 6-month follow-up, the significant difference in the dichotomization of Glasgow Outcome Scale scores between the progesterone and placebo groups was similar to that after three-month injury (*P *= 0.048).

The 3-month and 6-month GOS scores between the progesterone and placebo groups are summarized in Table 2. There was a better recovery rate for the patients who were given progesterone than for those in the control group at 3-month follow-up (*P *= 0.044). A dichotomized analysis revealed significant differences in neurologic outcome between the treatment and control groups (Figure 2). The analysis using the dichotomization of GOS scores at 3 months post injury revealed a favorable outcome in 47% of the patients receiving progesterone and in 31% of the placebo group (*P *= 0.034). There was an unfavorable outcome in 53% of the patients receiving progesterone and in 70% of the placebo group (*P *= 0.022). At 6-month follow-up, the dichotomized GOS scores also showed a significant statistical difference between the two groups, similar to those 3 months after injury. The percentage of favorable outcome was 58% for the patients who were given progesterone and was 42% in the placebo group (*P *= 0.048). Forty-one percent of patients who were given progesterone and 57% of the placebo group exhibited an unfavorable outcome (*P *= 0.048).

**Table 2:** Comparison of Glasgow Outcome Scale scores between the progesterone and placebo groups patients at 3-month and 6-month follow-up

Glasgow Outcome Scale scores	Progesterone (n = 82)	Placebo (n = 77)
3 months		
Good recovery	21 (25)	10 (12)
Moderate disability	18 (21)	14 (18)
Severe disability	16 (19)	13 (16)
Vegetative survival	13 (15)	16 (20)
Death	15 (18)	25 (32)
6 months		
Good recovery	26 (31)	19 (24)
Moderate disability	22 (26)	14 (18)
Severe disability	9 (10)	11 (14)
Vegetative survival	10 (12)	8 (10)
Death	15 (18)	25 (32)

Data presented as n (%).

Subgroup analysis for women also showed a significant difference in the percentage of favorable outcome between the two groups at 6-month follow-up (35% in the placebo group and 66% in the progesterone group, *P *= 0.036). The patients who were given progesterone in the group with GCS of 6 to 8 showed a more favorable outcome (43%) compared with the placebo group (28%) at 6-month follow-up (*P *= 0.044). There was no significant difference, however, in dichotomized outcomes in the group with GCS of 3 to 5 (*P *> 0.05).

### Modified Functional Independence Measure scores

**Figure 3:**
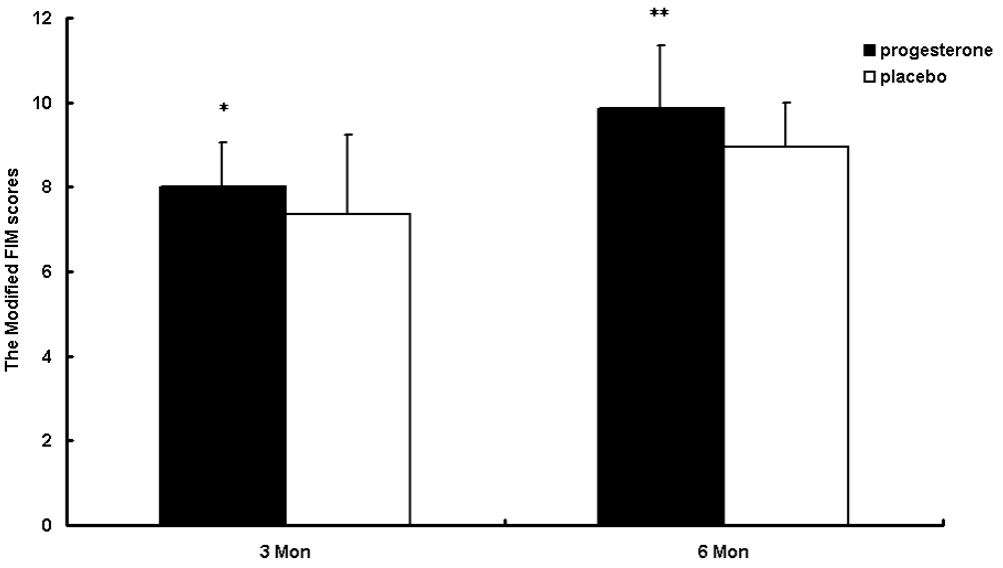
Modified Functional Independence Measure scores for patients receiving either progesterone or placebo. Modified Functional Independence Measure (FIM) scores at 3-month and 6-month follow-up from patients receiving either progesterone or placebo show that the scores in the progesterone group were significantly higher than those in the placebo group at both 3-month and 6-month follow-up. Data expressed as the mean ± standard deviation. Different from the placebo group: *P < 0.05, **P < 0.01.

Figure 3 shows the modified FIM scores at 3-month and 6-month follow-up. There was a significant difference in the mean modified FIM score between two groups both at 3-month and 6-month follow-up. At the 3-month follow-up, the scores were 7.35 ± 1.89 for the placebo group and 8.02 ± 1.73 for the progesterone group (*P *< 0.05). Six months after injury, the placebo group showed a score of 8.95 ± 1.05 and the progesterone group presented 9.87 ± 1.17 (*P *< 0.01), suggesting good functional outcome in the patients treated with progesterone.

### Mortality

During the 6 months of follow-up, a total of 40 patients (25%) died in the present study (37 patients died during their hospital stay). Seventy percent of deaths occurred within 1 week after trauma. Mortality was attributed to the heavy head injury in each case. The mortality rate in the progesterone treatment group was significantly lower at 6-month follow-up compared with the placebo group (18% versus 32%, *P *= 0.039).

### Intracranial pressure

**Figure 4:**
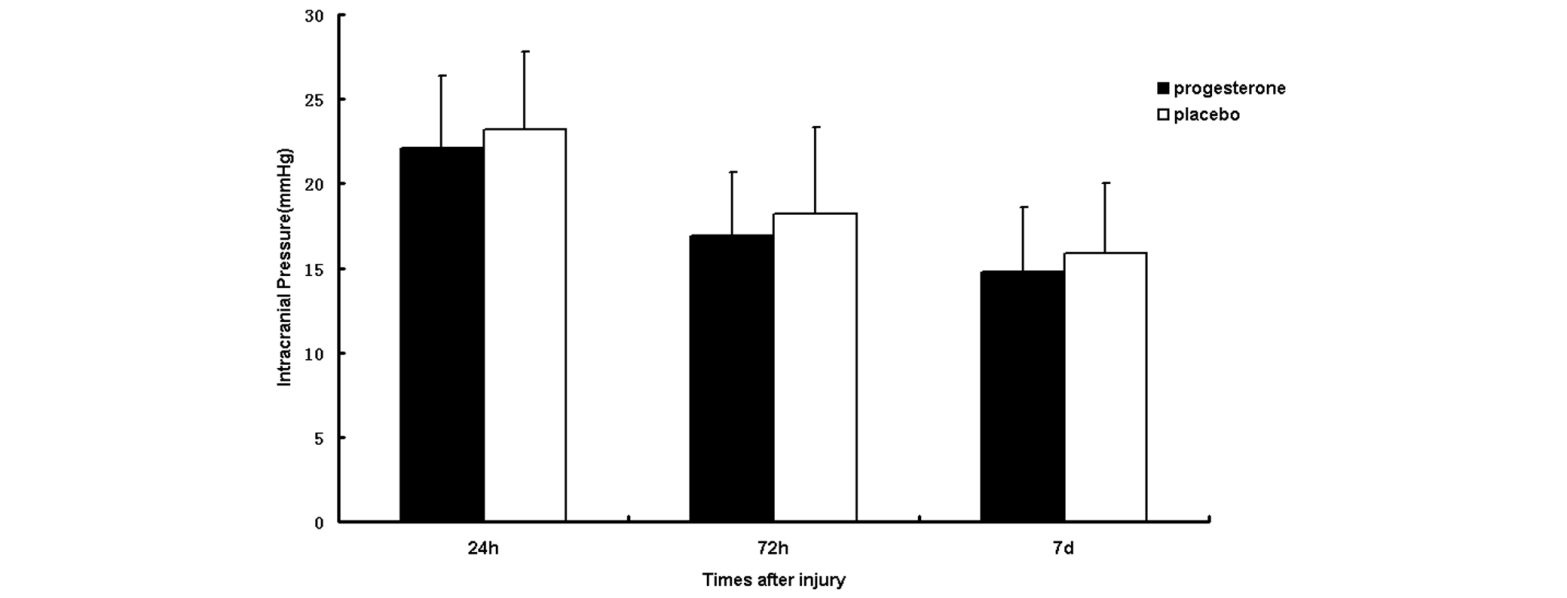
Comparison of intracranial pressure between patients receiving either progesterone or placebo. The mean intracranial pressure between the progesterone and placebo group patients shows no significant differences 24 hours, 72 hours and 7 days after injury between the two groups (*P *> 0.05). Data expressed as the mean ± standard deviation.

Figure 4 shows the ICP in the progesterone group patients and in the placebo group patients at 24 hours, 72 hours and 7 days after injury. The ICP was monitored continuously for 75 patients (47%), 40 in the progesterone group and 35 in the placebo group. The mean ICP shows no apparent difference at 24 hours after trauma between the two groups (progesterone group, 22.1 ± 4.3 mmHg versus placebo group, 23.2 ± 4.6 mmHg; *P *= 0.121). At 72 hours and 7 days after injury, the mean ICP of patients who were given progesterone was slightly lower than those of patients who received placebo, but the differences were not statistically significant (16.9 ± 3.8 mmHg and 14.8 ± 3.8 mmHg for progesterone-treated patients versus 18.2 ± 5.1 mmHg and 15.9 ± 4.1 mmHg for placebo-treated patients, respectively; *P *> 0.05).

### Glasgow Coma Scale scores and clinical measurements

The mean GCS scores increased progressively in the two groups during the 14-day acute phase of the study, with no apparent differences among the treatment groups. Meanwhile, there was no obvious difference in average body temperature, heart and respiratory rates, blood pressure, pulse blood oxygen saturation, and laboratory testing between the progesterone and placebo groups.

### Complications and adverse events

Progesterone was well tolerated in the treated patients with acute severe TBI. No complication and adverse event associated with the administration of progesterone was found in this clinical study during the hospitalization periods.

## Discussion

The results of the present trial showed for the first time that progesterone administration had a longer-term efficacy on clinical outcomes in acute TBI patients. A significant increase in the proportion of patients with a favorable outcome in the progesterone group compared with the placebo group up to 6 months indicates the possibility of progesterone for treatment of acute TBI. Moreover, there were more surviving TBI patients in the treatment group than in the control group. Our results suggest the efficacy of progesterone in the treatment of acute severe TBI.

Previous reports showed the evidence of efficacy in TBI animal models [8-14]. In the present study, the efficacy and safety of progesterone was confirmed in patients with acute severe TBI. Furthermore, our results using the modified FIM and GOS scores showed that progesterone administration remarkably enhanced functional recovery 6 months after injury and reduced the mortality of the patients with acute severe TBI (GCS = 6 to 8), although there was no statistical significance in the outcome improvement for GCS = 3 to 5 patients with and without progesterone treatment. The evidence of improved outcome for women patients also suggested, in part, a beneficial efficacy and feasibility of progesterone in women with TBI, in spite of the limited number of female patients in the trial.

It is recognized that the pathophysiology of TBI is a multifactorial process involved in a complex and interwoven series of pathologic process following the onset of insult, such as increased extracellular glutamate concentrations, increased intracellular Ca^2+^, free radical overproduction and exacerbated inflammatory response. Medication targeted at a pathological single injury factor could therefore not sufficiently recover functional deficits following TBI. The ideal drugs should be able to block multiple cellular events leading to brain damage following TBI. Neuroprotective strategies currently focus on acting on only one of the mechanisms. Some efforts have been made, however, to combine agents or interventions to increase the probability of success in this setting [23,24]. Nevertheless, the use of a single pharmacologic agent or procedure to slow or block damaging chemicals that are released after brain injury is highly desirable.

Progesterone has several features that make it an attractive potential drug candidate for TBI. First, progesterone could protect against brain damage via multiple mechanisms[13,15-18]. The pharmacokinetics of progesterone and its pattern of adverse reactions are well known since the drug has been safely used for a long time [25,26]. Second, with a wide therapeutic window of progesterone, a single bolus given up to 24 hours post injury may significantly reduce cerebral edema [7]. Third, progesterone may rapidly cross the blood–brain barrier and reach equilibrium with the plasma within 1 hour of administration [27-29]. Administration of progesterone soon after TBI would probably benefit the recovery of the patient.

In the present double-blind trial, progesterone or placebo was dissolved in the same camellia oil and taken daily for 5 days by patients with acute TBI. Those patients administered progesterone experienced significant improvements in functioning outcome, indicating neuroprotective properties of progesterone in acute severe TBI. There was no adverse event after administration of progesterone and no further late toxicity up to 6 months in the trial.

Goss and colleagues suggested that low and moderate doses of progesterone (4 to 8 mg/kg) were optimal for facilitating recovery of select behaviors, and that postinjury progesterone treatment permitted a wider dose range than preinjury treatment in rats with medial frontal cortical contusions [30]. In addition, 5 days of postinjury progesterone treatment are needed to reduce significantly the neuropathological and behavioral abnormalities found in a rodent model of TBI [13]. Wright and colleagues used intravenous progesterone at a dose of 0.71 mg/kg, followed by 0.5 mg/kg progesterone per 12 hours during the 3 following days, which appeared safe in the treatment of TBI patients [19]. In our study, the patients were received a single intramuscular injection of 1.0 mg/kg progesterone and the same dose per 12 hours for 5 consecutive days. The results in our trial showed that single higher-dose progesterone as protective therapy did not lead to any serious side effects. No obvious symptoms of hormone reaction were observed in our study. Accordingly, it can be anticipated that progesterone may be a promising treatment for severe TBI patients as it is inexpensive, widely available and has a long track record of safe use in humans to treat other diseases.

The data in the present study provide very encouraging and favorable conditions that could lead to the assessment of GOS and FIM scores in TBI patients in a clinical trial. The GOS score, although useful, provides only a global assessment of function and dependence; it may not differentiate specific difference in cognitive function, motor function, or daily activities. The modified FIM score selects only three items from the 18-item score, and also distinguishes only four (as opposed to seven) levels of function. Subtle or complex deficiencies, particularly in cognitive function, may not have been identified in the dataset. A deficiency in using any one scale to measure outcome is that it is limited in its scope of measurement. The present clinical study was therefore designed to evaluated functional outcome according to the GOS and the modified FIM score.

Intracranial hypertension has been considered an important factor affecting the outcome of the patients with acute severe TBI. Progesterone administration showed to decrease cerebral edema [9]. In an experimental study with male rats, there was a linear correlation between the serum progesterone level and brain edema after experimental TBI. The higher the serum progesterone level, the lower the cerebral edema [31]. In the current trial, however, no statistically significant difference was found in ICP monitoring between the groups given progesterone or placebo. It seems that progesterone treatment has little effect on directly reducing the ICP of patients with acute severe TBI.

As a result of randomization, all of these parameters were homogeneous between the progesterone and placebo groups in our clinical trial. Nevertheless, some limitations are observed in the current study. The results could be influenced by a single-center trial and local perioperative standard of care. Therefore, it is necessary to use a sufficient power to assess progesterone's effects on neurologic outcomes. Our result of the significant differences in outcomes between two groups of patients emphasizes the potential value of using GOS and FIM to tailor progesterone administration and the likelihood of observing similar differences in a larger patient population; however, the possibility exists that a statistical error may have occurred because of an inadequate sample size. Further studies are needed to determine the mechanisms of action underlying the neurologic effect observed.

## Conclusion

The present pilot study indicated that the use of progesterone may significantly improve neurologic outcome of patients suffering severe TBI up to 6 months after injury, providing a potential benefit to the treatment of acute severe TBI patients. Our results strongly support further large, multicenter clinical trials to examine the ways in which progesterone is achieving the profound neurologic effect and to decipher optimal conditions in which it can be used to lengthen the duration of and improve the degree of neuroprotection.

## Key messages

• Many neuroprotective agents have been shown to be efficient on TBI in animal models, and there is no single agent that shows improvement in outcome for head injury patients.

• A number of experimental models have suggested that administration of progesterone has a potential benefit for head injury.

• The present clinical trial reveals that progesterone may be used as a potential safe drug for the treatment of acute severe head trauma patients.

## Abbreviations

FIM = Functional Independence Measure; GCS = Glasgow Coma Scale; GOS = Glasgow Outcome Scale; ICP = intracranial pressure; TBI = traumatic brain injury.

## Competing interests

The authors declare that they have no competing interests.

## Authors' contributions

GMX and WQY participated in the trial design and were involved in the study analysis and summary. GMX and WMW obtained the data. GMX, JW, ZHL and WMW participated in the data analysis and interpretation of the results. All authors reviewed the final version.
